# Ghrelin plasma levels, gastric ghrelin cell density and bone mineral density in women with rheumatoid arthritis

**DOI:** 10.1590/1414-431X20175977

**Published:** 2017-05-18

**Authors:** F.A.N. Maksud, A.M. Kakehasi, M.F.B.R. Guimarães, C.J. Machado, A.J.A. Barbosa

**Affiliations:** 1 Departamento de Clínicas Pediátrica e de Adultos, Escola de Medicina, Universidade Federal de Ouro Preto, Ouro Preto, MG, Brasil; 2 Departamento do Aparelho Locomotor, Faculdade de Medicina, Universidade Federal de Minas Gerais, Belo Horizonte, MG, Brasil; 3 Serviço de Reumatologia, Hospital das Clínicas, Faculdade de Medicina, Universidade Federal de Minas Gerais, Belo Horizonte, MG, Brasil; 4 Departamento de Medicina Preventiva e Social, Faculdade de Medicina, Universidade Federal de Minas Gerais, Belo Horizonte, MG, Brasil; 5 Departamento de Anatomia Patológica, Faculdade de Medicina, Universidade Federal de Minas Gerais, Belo Horizonte, MG, Brasil; 6 Instituto Alfa de Gastroenterologia, Hospital das Clínicas, Universidade Federal de Minas Gerais, Belo Horizonte, MG, Brasil

**Keywords:** Ghrelin, Gastric mucosa, Bone metabolism, Osteoporosis, Rheumatoid arthritis

## Abstract

Generalized bone loss can be considered an extra-articular manifestation of rheumatoid arthritis (RA) that may lead to the occurrence of fractures, resulting in decreased quality of life and increased healthcare costs. The peptide ghrelin has demonstrated to positively affect osteoblasts *in vitro* and has anti-inflammatory actions, but the studies that correlate ghrelin plasma levels and RA have contradictory results. We aimed to evaluate the correlation between total ghrelin plasma levels, density of ghrelin-immunoreactive cells in the gastric mucosa, and bone mineral density (BMD) in twenty adult women with established RA with 6 months or more of symptoms (mean age of 52.70±11.40 years). Patients with RA presented higher ghrelin-immunoreactive cells density in gastric mucosa (P=0.008) compared with healthy females. There was a positive relationship between femoral neck BMD and gastric ghrelin cell density (P=0.007). However, these same patients presented a negative correlation between plasma ghrelin levels and total femoral BMD (P=0.03). The present results indicate that ghrelin may be involved in bone metabolism of patients with RA. However, the higher density of ghrelin-producing cells in the gastric mucosa of these patients does not seem to induce a corresponding elevation in the plasma levels of this peptide.

## Introduction

Rheumatoid arthritis (RA) is a systemic inflammatory disease that affects the synovial joints in the form of chronic polyarthritis (1). Decreases in bone mass and changes in body composition are common in patients with RA, especially in users of glucocorticoids and postmenopausal women. The prevalence of osteoporosis is higher than 50% in some RA patients, who have almost double the risk of vertebral and non-vertebral fractures compared with age- and gender-matched controls (2,3).

Bone erosion is the final consequence of the cartilaginous and bone tissue loss due to persistent local inflammation. Tumor necrosis factor alpha (TNF-α) and other inflammatory cytokines, such as interleukins (IL) 1, 6, and 17, are involved in this process by stimulating osteoclastogenesis and thus inducing bone erosion (4–6). In addition, bone tissue can be affected by the systemic inflammatory environment, by physiological influences, including hormones and growth factors, and by environmental influences, such as eating habits. Also, mechanical stresses can cause disruption of the remodeling bone process, leading to low bone mass with increased fragility and fractures (7).

The peptide ghrelin, a potent growth hormone secretagogue discovered in 1999, has been linked to many physiological and pathophysiological aspects in addition to growth (8,9). The production of this peptide occurs mostly in the endocrine cells of the oxyntic gastric mucosa, responsible for about 80% of its serum levels (10). More recently, studies in patients with RA and other inflammatory conditions have shown that activation of the immune system is accompanied by disturbances in energy homeostasis and in plasma ghrelin levels (11,12). *In vitro* studies show a possible action of this peptide in the differentiation and proliferation of osteoblastic cells in culture (13,14). Ghrelin has an anabolic effect on bone tissue and a positive relationship with the trabecular bone density (15). On the other hand, results of the effects of ghrelin on osteoclastogenesis are contradictory, and there are reports of increased bone reabsorption by ghrelin-stimulated osteoclasts in mice, although a change in the differentiation of osteoclasts in bone marrow has not been shown (16).

Plasma levels of ghrelin could also be related to inflammatory factors and autoimmune disease activity. The expression of ghrelin in lymphoid system suggests a role for this peptide and its receptor in the regulation of the immune system (17). Ghrelin has an anti-inflammatory action through downregulation of nuclear factor kappa B (NF-kB), thus increasing levels of nitric oxide in areas of inflammation and reducing expression of cytokines, particularly TNF-α. In an experimental model of RA, subcutaneous ghrelin administration reduced the levels of IL-6 and clinical signs of the disease (18,19). Otero et al. (20) observed lower levels of serum ghrelin in patients with RA in comparison with healthy controls, while Magiera et al. (21) showed reduced serum levels of ghrelin after treatment with infliximab.

As the peptide ghrelin is mainly produced by endocrine cells of the gastric mucosa, and seems to have a role in bone metabolism in patients with RA, we analyzed the ghrelin-producing cells in these patients, and correlated the results with ghrelin plasma levels, and also with the patient bone mineral density (BMD).

### Patients and Methods

Female adult patients with RA defined according to the classification criteria of ACR/European League Against Rheumatism (EULAR) 2010 (22) with 6 months or more of symptoms, were consecutively invited to participate. Patients with other diseases besides RA, smokers and those using bisphosphonates were excluded. We performed an active search of the files of 100 patients attending the endoscopy service of a university hospital and found twenty patients who had been submitted to bone densitometry and upper digestive endoscopy, with biopsies of the oxyntic and antral gastric mucosa within the last 3 months.

All participants were subjected to a complete clinical history, and body mass index (BMI) was measured as weight in kilograms divided by the square of height in meters (kg/m^2^). The RA activity was assessed by the disease activity score in 28 joints, using the DAS28 index (23).

Bone densitometry of the lumbar spine and proximal hip was measured by dual-energy X-ray absorptiometry (DXA; Discovery W; Hologic Inc., USA) and was analyzed by the same examiner. The results of BMD are reported in g/cm^2^. The BMD classification into normal, low bone mass, and osteoporosis was based on the World Health Organization (WHO) guidelines (24).

Blood samples were taken from peripheral vein after a 6-h fasting, and total plasma ghrelin was quantified by a sandwich enzyme-linked immunosorbent assay (ELISA) kit (Linco Research, USA) in accordance with the manufacturer's instructions. The inter- and intra-assay coefficients of variation were 5.2 and 1.1%, respectively.

Fragments obtained from gastric biopsies were routinely processed for embedding in paraffin and were stained with hematoxylin and eosin (HE) and Giemsa for histology and *Helicobacter pylori* detection. The biopsy specimens were examined in a blind manner by a pathologist and scored to indicate the presence and intensity of inflammatory infiltrate, glandular atrophy and intestinal metaplasia according to the updated Sydney system (25).

Ghrelin-immunoreactive cells were detected by immunohistochemistry using a polyclonal primary antibody against human ghrelin (Phoenix, USA). The density of ghrelin-immunoreactive cells was calculated by morphometric analysis using the KS 300 software (Zeiss, Germany) in three to five consecutive columns at 400× magnification with preserved mucosal areas. The results are reported in cells/mm^2^ in areas of oxyntic preserved mucosa.

For morphological comparison, a group of 20 healthy female patients were selected from the endoscopy service database, and were matched with RA patients, according to age, BMI, and macroscopic endoscopic findings. Patient records were assessed and those with inflammatory diseases or use of medications were excluded.

We applied the Student *t-*test and the Mann-Whitney test for continuous and paired samples. The univariate and multiple regression analyses was carried out using Stata Statistical Software (2011 release 12.0; , Stata Corp., Texas). The aim was to compare the BMD coefficients in the models with only plasma ghrelin or ghrelin cell density, and then with covariates. Graphs were built using GraphPad Prism software version 6.07 and Pearson correlation coefficients were obtained, given the linear relationship. A value of P<0.05 was considered to be statistically significant. The research project was approved by the UFMG Ethics Committee and all participants provided written informed consent.

## Results

Twenty female adult patients with RA were included, with a mean age and BMI of 52.70±11.40 years and 27.10±6.30 kg/m^2^, respectively. The DAS28 index was 3.88±2.09 and only 1 patient was not in a period of active disease. The mean age and BMI of the healthy group were 51.85±12.25 years and 24.5±4.8 kg/m^2^, respectively.

**Figure 1.**
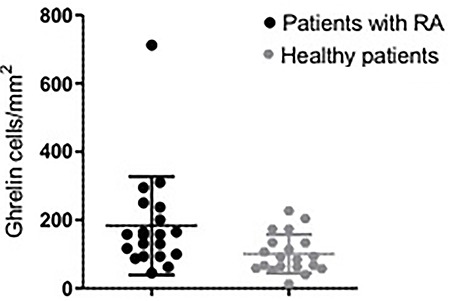
Comparison of the mean density and standard deviation of ghrelin-immunoreactive cells in patients with rheumatoid arthritis (RA) and in healthy patients (n=20). P= 0.008 (Mann-Whitney test).

**Figure 2.**
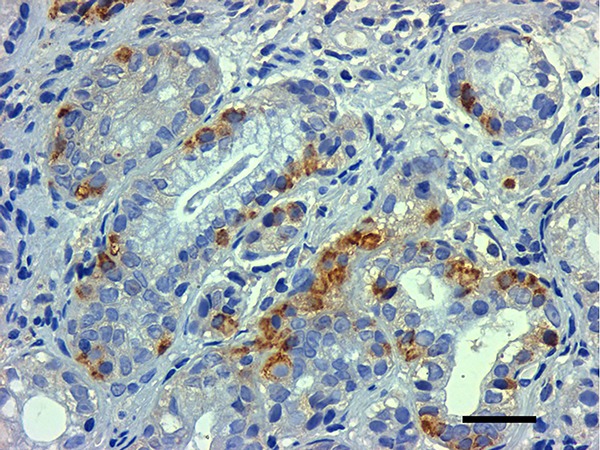
Immunostaining of ghrelin in gastric glands. Foci of linear hyperplasia of ghrelin-immunoreactive cells in the oxyntic mucosa of a patient with rheumatoid arthritis. Magnification: 400×. Scale bar: 35 µm.

Histological analyses showed mild chronic pangastritis in 12 (65%) RA patients, and in 14 (70%) healthy patients (P=0.207; Table 1). Gastritis with glandular atrophy was seen in 4 (20%) RA patients, and 3 of these presented atrophic body gastritis (ABG) compatible with gastritis of autoimmune origin. The mean density of ghrelin-immunoreactive cells in the oxyntic mucosa of RA patients was higher than in the healthy group (184.10±144.32 *vs* 100.81±57.32 cell/mm^2^, respectively; P=0.008; Figure 1). The oxyntic gastric mucosa of RA patients presenting ABG had diffuse endocrine cell hyperplasia, and many of these cells were immunoreactive to ghrelin with a mean density of ghrelin-immunoreactive cells of 364.60 cell/mm^2^ (Figure 2). Considering only RA patients without ABG, the difference remained significant in comparison with the healthy female group (152.30±75.81 *vs* 99.63±58.72 cell/mm^2^, respectively; P=0.046).

**Table 1.** Clinical, endoscopy features and densitometry characteristics of the study subjects.

	Patients with RA (n=20)	Healthy patients (n=20)	P value
Age (years)	52.74 ± 11.48	51.85 ± 12.25	0.68
BMI (kg/cm^2^)	27.1 ± 6.3	24.5 ± 4.8	0.37
DAS 28	3.88 ± 2.09	-	
Normal gastric mucosa (n/%)	4 (20%)	6 (30%)	0.207
Gastritis without atrophy (n/%)	12 (60%)	14 (70%)	0.207
Gastritis with atrophy (n/%)	4 (20%)	0	0.207
*H. pylori* infection (n/%)	4 (20%)	7 (35%)	
Osteoporosis (n/%)	4 (20%)	*	
Osteopenia (n/%)	13 (65%)	*	

DAS 28: disease activity score in 28 joints (Ref. 23); BMI: Body mass index; RA: rheumatoid arthritis.

* Not evaluated.

**Figure 3.**
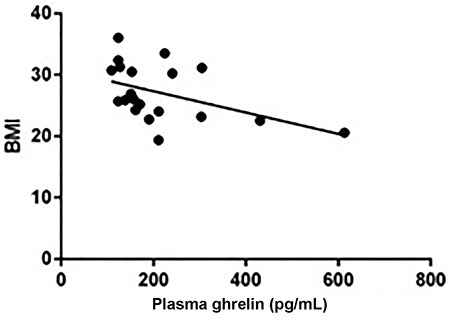
Correlation between plasma ghrelin levels and body mass index (BMI) in patients with rheumatoid arthritis (P=0.028; Pearson=-0.488).

There was no correlation between the density of ghrelin-immunoreactive endocrine cells and BMI in RA patients and the healthy comparative group (P=0.392 and 0.249, respectively). In addition, no correlation was found between the density of ghrelin-immunoreactive cells and plasma ghrelin levels in RA patients (P=0.465); however, the plasma ghrelin levels negatively correlated with BMI in RA patients (P=0.019; Figure 3).

The DXA scan results indicated that 4 (20%) RA patients presented osteoporosis and 12 (65%) presented osteopenia (Table 1). The average BMD of the RA patients was 0.620±0.330 g/cm^2^ in the lumbar spine, 0.520±0.170 g/cm^2^ in the total femur, and 0.660±0.160 g/cm^2^ in the femoral neck.

**Figure 4.**
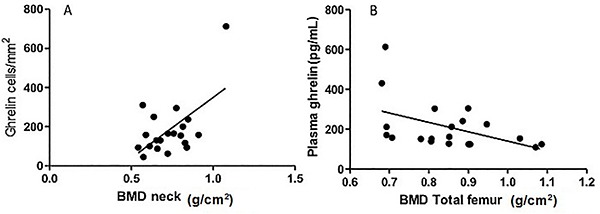
Positive correlation between the density of ghrelin-immunoreactive cells and bone mineral density (BMD) of femoral neck (P=0.007; Pearson=0.579) and negative correlation between plasma ghrelin levels and total femur BMD (P=0.03; Pearson=-0.4694) in a group of twenty women with established rheumatoid arthritis.

In univariate graphic analysis, a positive correlation was found between the femoral neck BMD and the density of ghrelin-immunoreactive cells in RA patients (P=0.004), and a negative correlation was found between femur total BMD and plasma ghrelin levels (P=0.038; Figure 4). In multivariate analysis, after adjustment with other covariates, the parameters that were found to be associated with BMD were age and BMI (Table 2).

**Table 2.** Multiple regression analysis of bone mass density in 20 patients with rheumatoid arthritis adjusted for the influence of covariates.

Variables	BMD L1-L4		BMD total femur		BMD femoral neck	
	Coef	P value	Coef	P value	Coef	P value
Age (years)	-0.011	0.074	-0.000	0.018	-0.005	0.029
BMI	0.030	0.021	0.022	0.000	0.016	0.003
Plasma Ghrelin (pg/mL)	0.000	0.087	0.000	0.734	-0.000	0.735
Ghrelin cells (cells/mm^2^)	-0.007	0.126	-0.000	0.195	0.000	0.121
Glucocorticoid (mg)*	0	0.569	0	0.780	0	0.294
	R^2^: 0.388		R^2^: 0.610		R^2^: 0.790	
	F: 1.781		F: 6.957		F: 10.554	

BMD: bone mineral density; BMI: body mass index. R^2^: coefficient of determination; F: Fisher test.

* Accumulated dose.

## Discussion

In the present study, we demonstrated that the density of ghrelin-immunoreactive cells was high in the oxyntic gastric mucosa (the main production site of this peptide) of RA patients. This phenomenon may be an attempt of the body to correct the persistent inflammation or may be an adaptive event to a possible hypergastrinemia, which has been described in studies of RA patients (26). Gastrin receptors are found in ghrelin-producing cells, and there is a stimulatory effect of gastrin on the endocrine cells in the oxyntic mucosa. However, De Witte et al. did not demonstrate a correlation between serum gastrin and duration or activity of RA, or use of anti-rheumatic drugs (27,28).

The high density of ghrelin-immunoreactive cells in patients with RA may have been influenced by the three patients who simultaneously had ABG. Probably from autoimmune origin, patients with ABG have hyperplastic proliferation of neuroendocrine cells in the gastric atrophic mucosa; however, the difference remained statistically significant even after excluding the cases with glandular atrophy. It has been reported that numerous ghrelin-immunoreactive cells are often found in the endocrine hyperplasia in ABG (29), and ABG is more frequent in patients with RA than in the general population (30).

Pathological studies have not found a significant change in the gastric mucosa in RA, beyond of what is found in patients taking anti-inflammatory drugs and in the presence of *H. pylori* (31). Patients with RA included in this study had similar histological findings to the healthy group, although some patients with RA had findings suggestive of glandular atrophy typical of autoimmune gastritis. Atrophic gastritis, a hypochlorhydric condition, has been linked to low bone mass, although we were not able to show a correlation between autoimmune gastritis and decreased BMD in postmenopausal women in a previous study (32,33).

An important finding was that the cell density in the gastric mucosa was higher in patients with higher mineral density in the femoral neck. These results are in agreement with Gonnelli et al. and Napolli et al. (14,15), who showed a positive correlation between plasma ghrelin levels and BMD in the femoral neck. Coates et al. (34) found a significant decrease in serum ghrelin levels and BMD in patients undergoing gastrectomy for bariatric surgery, supporting the importance of the stomach endocrine cells in BMD.

Similar to results of previous studies (35,36), the plasma ghrelin levels were not associated with lumbar or femoral neck BMD. However, ghrelin levels were negatively associated with total femur BMD, similar to the results reported by Weiss et al. (37) in postmenopausal women. It is important to mention that ghrelin plasma levels may be affected by other hormones, such as gastrin and leptin (27,38), and especially by age, as reported by Nouh et al. (39) in a study of pre-, peri- and postmenopausal women, which found a significant positive correlation between plasma ghrelin level, estradiol and BMD. Jürimäe et al. (40) demonstrated that the association between plasma ghrelin levels and bone density is influenced by body composition. These data could partially explain the negative correlation between plasma ghrelin levels and total femur BMD in univariate analysis but not in multivariate analysis. Because of the small sample size, participating women were not stratified by menopausal period categories or by body composition, and this was a limitation of the present study. Another limitation was the impossibility to accomplish the DXA in the healthy group and quantify their plasma levels of ghrelin.

Several molecular studies have demonstrated an anabolic action of ghrelin on osteoblasts and chondrocytes (13,16,37), and the present study is the first to correlate the density of ghrelin-immunoreactive cells with BMD and with the presence of RA. We conclude that the peptide ghrelin played a role in bone formation and control of inflammatory conditions such as RA. However, local and indirect effects of this peptide are still uncertain and more studies are needed to better understand these effects.
